# Transcranial electrical stimulation: How can a simple conductor orchestrate complex brain activity?

**DOI:** 10.1371/journal.pbio.3001973

**Published:** 2023-01-30

**Authors:** Matthew R. Krause, Pedro G. Vieira, Christopher C. Pack

**Affiliations:** Department of Neurology and Neurosurgery, Montreal Neurological Institute, McGill University, Montreal, Quebec, Canada

## Abstract

Transcranial electrical stimulation (tES) is one of the oldest and yet least understood forms of brain stimulation. The idea that a weak electrical stimulus, applied outside the head, can meaningfully affect neural activity is often regarded as mysterious. Here, we argue that the direct effects of tES are not so mysterious: Extensive data from a wide range of model systems shows it has appreciable effects on the activity of individual neurons. Instead, the real mysteries are how tES interacts with the brain’s own activity and how these dynamics can be controlled to produce desirable therapeutic effects. These are challenging problems, akin to repairing a complex machine while it is running, but they are not unique to tES or even neuroscience. We suggest that models of coupled oscillators, a common tool for studying interactions in other fields, may provide valuable insights. By combining these tools with our growing, interdisciplinary knowledge of brain dynamics, we are now in a good position to make progress in this area and meet the high demand for effective neuromodulation in neuroscience and psychiatry.

## Abstract 2

Weak electric currents applied to the scalp (known as transcranial electrical stimulation) can affect brain activity in surprisingly complex ways. This Unsolved Mystery investigates how these effects can be used to treat disease or improve cognition.

## Introduction

In 46 AD, the Roman physician Scribonius Largus reported that headaches and chronic pain could be cured by placing a certain kind of fish near the affected area [1]. Similar prescriptions appeared in medical texts for hundreds of years, but not everyone believed them: Galen, one of the most prominent physicians of the second century, wrote that the fish in question “is said by some to cure headache and prolapsus ani. I indeed tried both of these things and found neither to be true.” Even its advocates could not agree on a mechanism of action: popular theories included magic, cold, and poison. Without an understanding of the mechanisms behind the fish’s therapeutic effects, ancient physicians struggled to determine if, when, how, and for whom treatment would be effective.

We now know that these fish, known as Atlantic Torpedoes, emit electrical pulses to stun their prey. Scribonius had accidentally repurposed these defensive discharges to alter the activity of his patients’ nervous systems. In the 1700s, discoveries revealed that the brain uses electrical impulses to transmit and process information. Shortly thereafter, researchers, clinicians, and charlatans all began exploring whether electricity could treat diseases, improve mental performance, or experimentally perturb brain function. Recent years have seen a surge of interest in this technique, now called transcranial electrical stimulation (tES).

**Fig 1:**
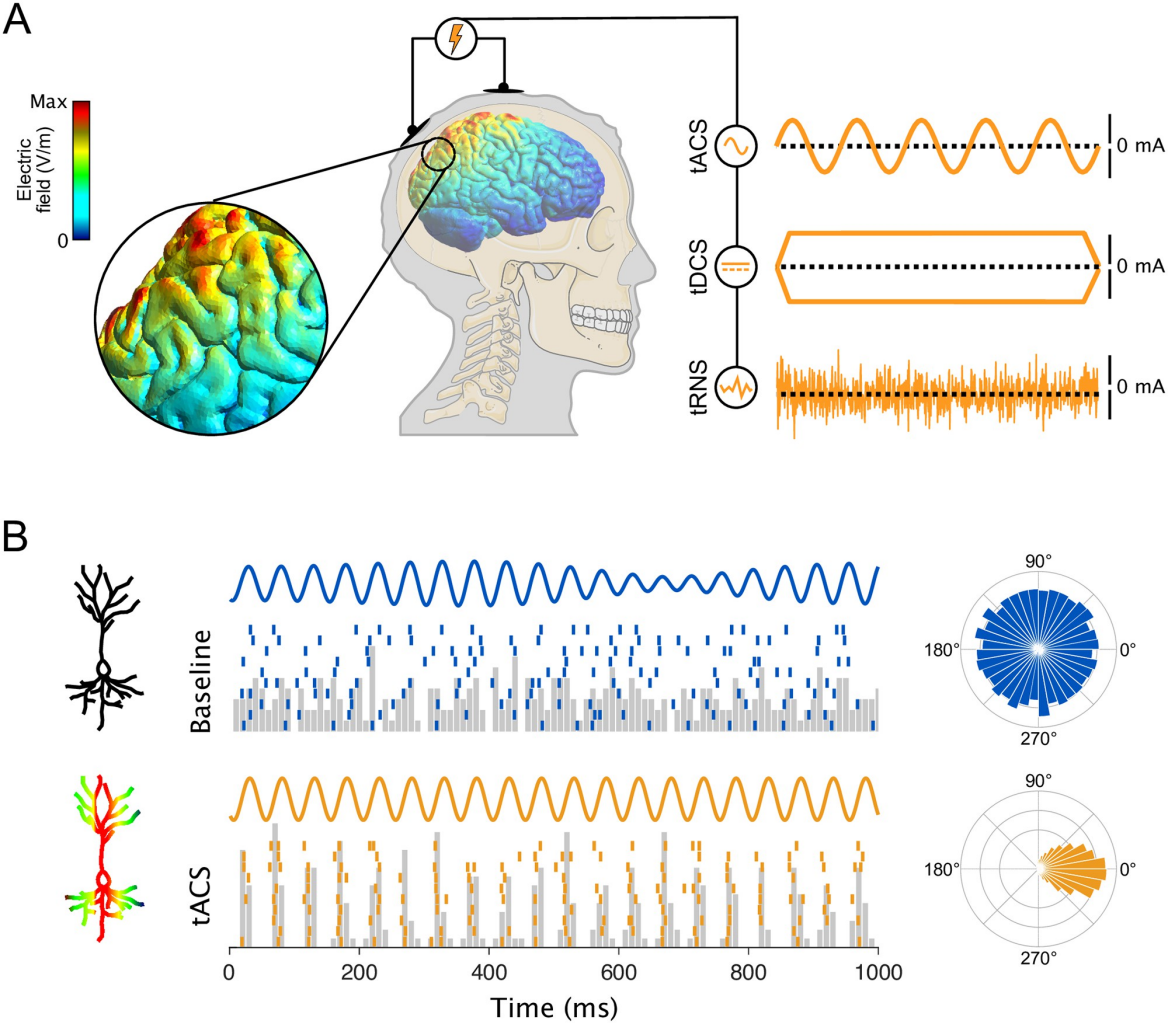
Modern tES uses battery-powered stimulators to alter brain activity. ** (A)** To create intracranial electric fields, weak currents are applied to the user’s scalp. Different types of tES can deliver as different current waveforms, including tACS, tDCS, and tRNS. **(B)** Despite some early skepticism, it is now clear that these fields do affect the activity of individual neurons. Each tick indicates the time of an action potential recorded from a basal ganglia neuron in an awake monkey. Without stimulation, the neuron’s activity is disorganized and arrhythmic; the timing of the spikes is unrelated to the phase of an ongoing brain oscillation (blue trace). During tACS, the neuron becomes entrained to the stimulation and fires during a specific part of its cycle. The average activity over ten 1-s segments is shown in gray. The preferred phases of the neuron in each condition are shown in the polar histograms (right). See [4] for experimental details. The head in Fig 1 was adapted from a CC-BY 3.0 licensed illustration by Servier/BioIcons. The electric fields in the brain were generated with SimNIBS [5]. tACS, transcranial alternating current stimulation; tDCS, transcranial direct current stimulation; tES, Transcranial electrical stimulation; tRNS, transcranial random noise stimulation.

In its modern form, tES uses a battery-powered stimulator to pass weak (1 to 4 mA) electrical currents between electrodes attached to the scalp, as shown in Fig 1A. Various forms of tES are distinguished based on how the current flows. One of the most common forms of tES is transcranial direct current stimulation (tDCS), which applies a constant current that continually flows in the same direction, from one electrode (the anode; see S1 Glossary) towards another (the cathode), creating a static electrical field. Another common variant, transcranial alternating current stimulation (tACS), instead applies current that regularly alternates directions, creating an electric field that oscillates. More exotic waveforms, including transcranial random noise stimulation (tRNS), square or triangular pulses, or those mimicking brain activity, are also sometimes used.

Some studies using these modern devices report dramatic improvements in the symptoms of devastating psychiatric conditions, like major depressive disorder. However, these results often prove difficult to replicate and extend, hindering their transition from the lab to the clinic. For example, a large meta-analysis recently concluded that existing data demonstrate “probable” or “possible” benefits of tES for depression and chronic pain, while cautioning that these studies do not yet provide the “definitive evidence” needed to make it part of routine clinical practice [2]. Promising but not decisive neuroenhancement results have also been reported in healthy users [3]. Thus, although we have come a long way from the fish-based treatments of the Romans, concerns about reproducibility and mechanism still limit the application of tES.

Ongoing work from many labs is attempting to demystify why and how external electrical impulses affect behavior. Most recent attempts have overwhelmingly focused on one question: Are the electric currents used during tES strong enough to affect brain activity? This is certainly an important question, but one that we believe has now been answered thoroughly. Convincing evidence demonstrates that tES, as used in humans, does indeed alter neural activity. Armed with these data, the more pressing question is how can we harness this knowledge to produce long-lasting, beneficial effects on the brain? This is the unsolved mystery of modern tES.

## Yes, tES does affect brain cells!

To be useful, any form of brain stimulation must somehow affect the spiking activity of neurons, the currency of communication within the brain. Somewhat alarmingly, there have been suggestions that tES does not even meet this minimal criterion [6–8], because much of the electrical current is shunted through the skin and never reaches the brain at all. This has led to a search for alternate explanations for the apparent influence of tES on behavior, including placebo effects, off-target stimulation (e.g., of the skin), and poor research practices. These kinds of claims have generated a great deal of skepticism about the technique, and consequently, its potential effects on behavior have been regarded as somewhat mysterious.

It might therefore surprise some readers to know that studies directly measuring brain activity overwhelmingly support the idea that tES alters the spiking activity of neurons. In fact, studies in isolated brain slices, e.g., [9,10]; rodents, e.g., [11]; ferrets, e.g., [12]; and non-human primates [4,13–15] have largely converged on the specific finding that tES alters the timing, but not the rate, of single-neuron spiking activity at field strengths found in human brains [16,17].

The effectiveness of tES is most evident in the application of tACS (Fig 1A). Neurons become entrained to the sinusoidal currents, shifting their spikes towards certain phases of the sinusoid’s waveform and away from others (Fig 1B). Control experiments have shown that this entrainment occurs independently of stimulation of peripheral nerves in the skin [14] or the retina [4], even though stimulating these structures sometimes produces similar effects. A related criticism is that spiking activity only changes once the electric field reaches a certain minimum strength. However, there is no plausible mechanism that would impose such a threshold, and direct measurements indicate that electric fields exert a graded, linear effect on neural activity [6]. Thus, we do not consider the ability of tES to influence individual neurons to be a mystery.

## How does tES affect brain circuits?

Lone neurons rarely affect behavior. Instead, each contributes to the activity of larger brain circuits responsible for perception, cognition, and action. Neural activity within these circuits creates structured patterns of activity, often in the form of oscillations in which large groups of neurons fire together rhythmically. Many oscillations are so widespread that they can be measured non-invasively on the scalp with electroencephalography (EEG), allowing oscillations to be linked with behavioral and clinical states. For example, within a single individual, moment-to-moment changes in the so-called “alpha” frequency band (8 to 12 Hz) of the EEG have been associated with changes in attention and arousal. Faster oscillations, in the 16 to 24 Hz “beta” band, instead reflect changes in a person’s motor preparation or control. Oscillations also differ between patients and healthy controls in the same behavioral states, suggesting that changes in oscillatory activity may be implicated in various clinical conditions as well.

Recreating desirable patterns of brain activity may therefore provide a way to restore—or even enhance—normal brain function. Like most brain oscillations, tES is structured in time (Fig 1B) but diffuse in space (Fig 1A), and some forms of it, like tACS, produce frequency-specific changes in spike timing. Thus, it could potentially replace an oscillation that is somehow malfunctioning. This is the logic behind most current tACS interventions. For example, Marchesotti and colleagues observed weakened 30 Hz oscillations in the left auditory cortex of patients with dyslexia as compared to controls [18]. They therefore applied 30 Hz tACS to that location, after which patients’ reading performance improved. They interpret these results as demonstrated that tES successfully reinstated the dyslexia-impaired 30 Hz oscillation, and therefore brought the patients’ brains closer to a healthy state.

Similar efforts are now underway in several countries to develop tES as a treatment for a wide range of medical conditions, including depression, schizophrenia, and epilepsy [19,20]. Early work in this area seems promising, but studies sometimes find that the same stimulation produces different, or even opposite, effects. For example, 10 Hz tACS increased frontal lobe alpha power in people with schizophrenia [21], but decreased alpha power in patients with major depressive disorder [22]. While neither study met its symptomatic end points, there was a trend towards reduced symptoms in both groups, suggesting tES may usefully alter brain activity.

But the question of mechanism continues to loom large. How could the same intervention produce opposite effects on brain activity—and improve brain function in both cases? The answer may lie in the preexisting patterns of brain activity. Patients with schizophrenia have abnormally small alpha oscillations while depression is associated with pathologically large ones. Since the direct effects of tES are relatively weak, it often cannot completely replace ongoing brain activity, but must instead interact with it in some way.

A recent study demonstrates how these state-dependent effects may occur [15]. Krause and colleagues recorded the activity of individual neurons in non-human primates receiving tACS, under conditions that closely matched human use. When ongoing oscillations were weak, they found that tACS successfully entrained neurons, as shown in Fig 1B. Surprisingly, applying the same stimulation when ongoing oscillations were stronger instead led to a paradoxical decrease in oscillatory spiking. Since even stronger stimulation could subsequently re-entrain these neurons (though at a different phase), they interpret these data as showing that tACS and ongoing brain activity compete for control over when a neuron spikes. The same stimulation could therefore cause categorically different neural effects due to the state of the brain, just as in the clinical studies described above.

Natural variations in brain state may therefore limit the effectiveness of tES. Ongoing oscillatory activity varies between individuals, in ways related to age, clinical conditions, and sex. Even within an individual, factors such as attentiveness and behavioral goal affect the oscillatory state of the brain and thus, its response to tES. Moreover, these oscillations are produced by interactions between complex networks at scales ranging from intracellular compartments to synaptic networks spanning the entire brain. Mathematicians have known for centuries that the dynamics of such complex systems can be difficult to predict and that they often react to perturbations in startling ways [23,24]. As a result, state-dependent effects of brain stimulation abound (reviewed in [25]), and so we cannot simply expect tES to impose a stimulation waveform directly onto the activity of a dynamic, living brain.

## An aside on complex systems theory

The problem of controlling complex systems is hardly unique to tES or even to neuroscience. It was described as a general challenge for science by Warren Weaver in 1948 [26] and in the context of economics by Friedrich Hayek in his 1945 essay “The Use of Knowledge in Society” [27]. Hayek claimed that economic theories are limited to what can be measured, and that these measurements might not be relevant to the overall health of the economy. Consequently, he concluded that economists often lack the ability to predict the outcome of particular policies. (This argument has subsequently been used to justify certain political causes, which we certainly are not promoting here.)

These problems might seem familiar to practitioners of tES: We can easily measure neural oscillations, but we can seldom be sure if they are causally related to specific brain functions. We can deliver controlled interventions, but often cannot precisely predict their effects. According to both Weaver and Hayek, these problems exist in any system that exhibits “organized complexity”—behaviors that depend on the precise properties of the system’s components and their interactions. Weaver drew a contrast between systems with “organized complexity” and those exhibiting “simple” dynamics or “disorganized complexity,” where only the behavior an individual component (simple) or their overall average behavior (disorganized complexity) is of interest.

**Fig 2:**
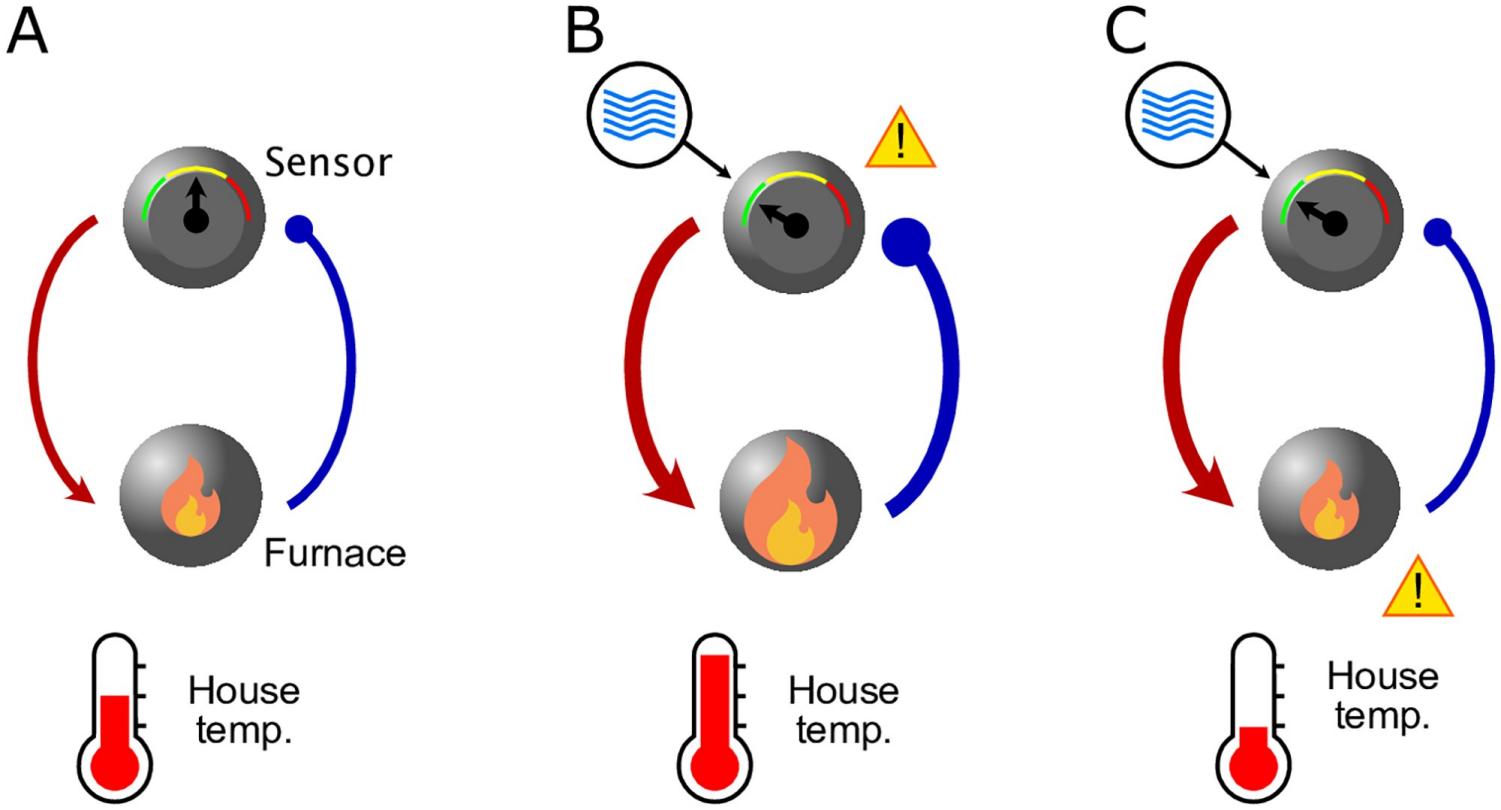
Internal dynamics can cause very simple systems to respond differently to the same intervention. **(A)** Suppose a heating system, comprised of a thermostat sensor and furnace malfunctions and the house is too cold. **(B)** If the sensor is defective, opening a window near the sensor might “stimulate” it, thereby increasing the furnace’s output and warming the house. **(C)** However, if the furnace is broken, this cools the house instead.

As an intuitive example of this complexity, consider a heating system that maintains a house at a particular temperature (Fig 2A). It has 2 interacting parts: a furnace that generates heat and a thermostat sensor that adjusts the furnace’s output. Now, suppose the system malfunctions and it becomes too cold in the house. What is the appropriate intervention for someone who does not have access to the individual components of this system? One might guess that the problem is the sensor: it is overestimating the temperature. In that case, a simple intervention is to open a window near the sensor, letting in some cold air (Fig 2B). Locally decreasing the temperature near the sensor will then trigger an increase in the furnace’s output, thereby warming the house globally. However, if that intuition is wrong and the problem is a faulty furnace instead, opening a window simply allows more cold air into the house, decreasing the temperature throughout (Fig 2C). Thus, the same intervention (opening a window) for the same observation (low indoor temperature) can have opposite effects because of a system’s internal dynamics.

A heating system has simple dynamics, but for more complex systems, the results of different interventions can defy intuition. This is true even when the components and connectivity are known [28]. As a result, interventions aimed at producing a particular goal often produce unpredictable or even perverse outcomes. Examples abound in the social and biological sciences: Rent control policies often decrease housing supply; designating a species as endangered can increase market demand for the protected animals. A humorous variant is the so-called Streisand effect, named after the singer whose attempts to suppress a photograph attracted publicity, causing the same photo to be widely shared.

Despite Hayek’s pessimism and many examples of failures to control complex systems, the example of the thermostat shows that it is possible—at least sometimes—to understand them. At a minimum, one needs a mechanistic model of the dynamics of the system and its responses to external inputs. The heating system seems tractable in this regard. So, is tES more like economics or home heating?

## How can one control a complex system?

The analogy between tES and an artificial system like a thermostat has obvious limitations. For example, a technician who wanted to fix a heating system would probably start by turning it off, permitting a careful inspection and adjustment of the individual parts. Nothing similar can be done with a living brain, so any attempt at neuromodulation must consider the organized complexity of brain’s internal dynamics and their response to the stimulation.

Weaver suggested that data and computation are the keys to addressing such complexity, and for tES, there is certainly no shortage of possible kinds of data, even just considering signals that can be detected with EEG [29]. From a single EEG electrode, one can extract the power and phase at every frequency band, as well as the envelope of its changes through time. Any one of these signals can maintain a specific relationship with signals recorded from nearby or distant brain areas, and such local or long-range coherence is often also considered a type of signal in its own right. Some tES applications attempt to influence these signals [30–32].

In most cases, the experimenter sets the stimulator to produce output resembling the desired effect on a particular brain area, based on a preexisting hypothesis about how that region should behave. The tES is then applied in an open-loop configuration, which does not adapt it in response to changes in its neural or behavioral effects. This approach can alter neural activity, but it is not difficult to see how it can lead to variable results: Oscillation frequencies are different for different subjects, and even small errors in estimating the appropriate frequency can lead to categorical changes in the influence of tES [15]. Moreover, the appropriate frequencies often change, so that even an initially well-designed protocol might wane in effectiveness over time. Perhaps most importantly, the brain’s own dynamics powerfully influence the response to stimulation. As a result, open-loop stimulation necessarily contains many hidden sources of variability.

Further technological advances could allow for closed-loop tES devices. These would act much like sophisticated thermostats, continually measuring neural activity and adapting stimulation parameters to produce a desired brain state. However, building such a system is a daunting engineering task. EEG recordings made during stimulation will contain both real neural signals and stimulation artifacts, as well as environmental noise, often within the same frequency band. Efforts are currently underway to develop methods for isolating brain activity from these signals (e.g., [33]), but there is not yet a clear consensus about the best way to do so [34]. On top of this, the system must respond quickly to changes in brain activity, which itself changes in response to stimulation and external events.

Even if these issues were resolved, the problem remains that, in the absence of a mechanistic model of the relevant brain function, each tES intervention might be valid only for the individuals and conditions on which the algorithms were trained; other conditions or users might require different control policies. We therefore suspect that improved technology alone will not be sufficient.

**Fig 3:**
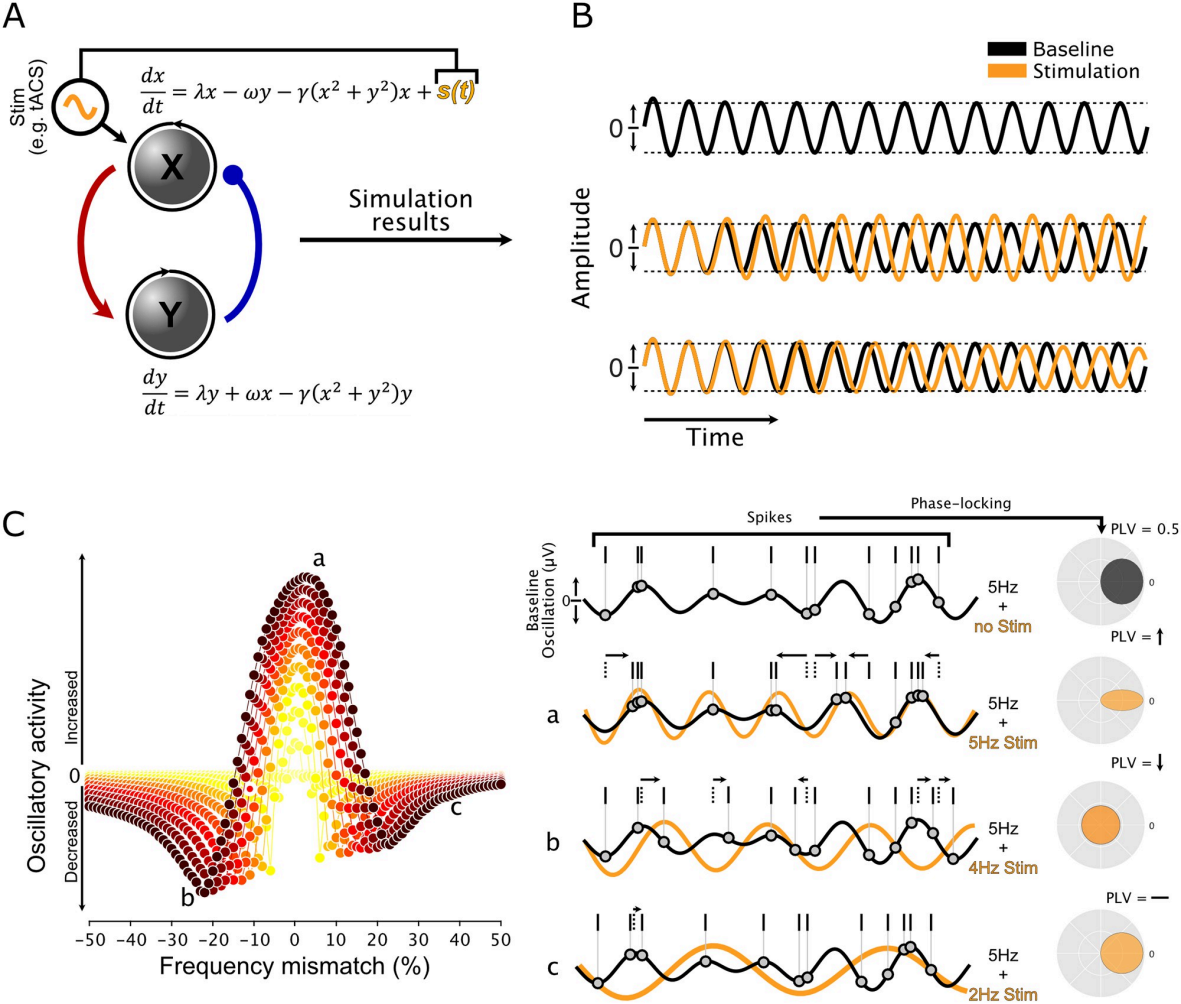
Simple oscillator models can account for the varied effects of tES. **(A)** The Stuart–Landau oscillator model consists of 2 interacting populations, much like the sensor-furnace model in Fig 2. Without external stimulation, the model oscillates continuously at a fixed frequency and amplitude (top). Applying a sinusoidal input to one population can increase (middle) or decrease (bottom) the oscillation’s amplitude, depending on the strength of the ongoing oscillation. See [12,27] for more details. **(B)** Interactions between the stimulation intensity and frequency relative to the ongoing oscillation can therefore generate a multitude of outcomes (left). The same pattern is evident in our neural data (right), where some combinations (a) increase entrainment between spikes and the EEG (b) decrease it, or (c) have no effect in that frequency range. Panel B adapted from [14]. EEG, electroencephalogram; tES, Transcranial electrical stimulation.

Instead, we suggest that the next step should be the development of tractable models of brain dynamics that predict the outcome of particular interventions and help select the desired outcome for specific goals. Producing useful models of brain function is no simple task, but the goal should not be to produce a detailed account of the underlying biology. We simply need to capture interactions between the relevant brain signals and tES. For example, a simple mathematical model of coupled oscillators [15] (Fig 3A) provided insight into the often unintuitive interaction between an oscillator with its own dynamics (such as the brain) and an external input that also oscillates (the stimulation). That model has only a few free parameters and requires less than a second to simulate with a standard laptop. Indeed, it is not much more complicated than the furnace example of Fig 2. Nevertheless, it qualitatively reproduced the full range of tACS’s effects on neural activity (Fig 3B), including several categorically different regimes.

Using such a model, it is possible to predict the effects of tES before applying the stimulation, simply by collecting some basic diagnostic information, which is also what an HVAC technician would do! Specifically, by measuring each participant’s peak oscillation frequency and the power at that frequency, one could derive parameters that would either increase an oscillation’s amplitude, decrease it, or shift the timing of spikes within a cycle.

The mathematics of coupled oscillators is, to the best of our knowledge, seldom used in studies of tES, but we and a few others [35] consider this to be a potentially fruitful direction for future research. Coupled oscillators are a well-developed set of mathematical tools that can nevertheless exhibit complex behaviors and are frequently used as models in other areas of science and medicine [36,37]. A promising approach might therefore be to compare large datasets of neural recordings to the properties of different mathematical oscillators in order to find models and parameters that capture the effects of tES. For example, many oscillators exhibit an “Arnold Tongue” [38]: a narrow range of parameters where stimulation enhances ongoing oscillations (see Fig 3B). However, the size and shape of this region, as well as the presence of suppressive flanks, varies between models [15]. Candidate models that could be easily interrogated to predict the likely effects of stimulation could provide a roadmap for future experiments. A recent example of this approach [39] used Kuramoto oscillators to successfully predict a patient’s response to deep brain stimulation (DBS).

One immediate suggestion from these models is that stimulation should be tailored to each individual user. Many studies already adjust the stimulation amplitude and/or location of tES electrodes to control for anatomical variations (e.g., skull or brain shape) that affect how the current flows through the head (e.g., [5,40]). This ensures that the resulting electric fields remain consistent across participants, an improvement over merely matching the stimulators’ outputs. It may be equally important to consider individual differences in physiology. Each user has their own idiosyncratic brain rhythms, which may interact differently with a fixed stimulus protocol. Preliminary data suggest that doing so can increase the reliability and efficacy of tES effects (e.g., [3]).

One advantage of oscillator models is that they provide a unifying framework for accounting for many factors that may affect tES. In addition to considering individual differences in rhythms, they can also be extended to including varying brain state, especially when viewed as a physiological factor rather than a behavioral one. For example, it may seem mysterious that tES has different effects when a recipient’s eyes are open or closed. However, knowing that this happens because eye closure increases alpha power reveals the similarity between this effect and the depression/schizophrenia difference described above. Similar approaches could be used to include factors like disease state and concurrent treatment with medication, which alter brain dynamics and may therefore also change the effects of stimulation.

Future work should also extend the models to areas outside the focus of stimulation. The electric fields produced during tES generally cannot directly affect firing rates, especially at the current levels matching human use, but theoretical work suggests that synchronizing neurons in one area can potentially influence both synchrony and firing rates in connected regions [41]. If true, this would allow one to tailor stimulation protocols to produce an even wider range of neural effects. All of these predictions, of course, still must be validated experimentally.

## What about other forms of tES?

While all forms of tES are somewhat mysterious, in our opinion, tACS is the closest to being understood. There is a clear hypothesis about how it acts—adjusting spike timing in a frequency-specific manner—and the neurophysiological data overwhelmingly agree that it does so. A hundred years of EEG research suggests potential targets for stimulation, and the well-developed theory of coupled oscillators provides a principled mathematical framework for predicting how brain oscillations and tACS might interact. Thus, we believe that tACS is currently the most tractable form of tES to study.

Other forms of tES seem more mysterious, but similar approaches may help understand them. For example, tDCS applies constant current to the scalp. This is often said to polarize neurons, causing those near the anode to become more excitable. This is certainly true biophysically and is supported by evidence from in vitro studies using high field strengths. However, when applied to living brains, the effects are more complicated: Sensory responses and synapses are modified, but firing rates remain largely unchanged [42–44]. Moreover, it is not clear how increasing overall neural excitability would affect a living brain. Excitatory and inhibitory neurons often interact to maintain stable firing rates [45–47]. Increasing excitability in both groups might therefore be expected to yield no net effect on firing rate, but could cause oscillations or other changes in spike timing, which have indeed been observed during tDCS [42]. As with tACS, understanding the effects of tDCS likely requires understanding the dynamic interactions between neurons and the stimulation. Models proposed for sensory systems, which also maintain homeostasis despite massive changes in input, may provide a useful starting point.

The situation with other forms of stimulation, such as tRNS, is even less clear. Noise is thought to produce “stochastic resonance” that amplifies weak signals by occasionally pushing them over a neuron’s spiking threshold. Despite promising behavioral findings [48,49], no in vivo physiological data is yet available and it is not obvious how this effect generalizes to networks of neurons with their own dynamics.

## How can we produce durable effects?

The approaches described above cause immediate changes in brain activity. Some potential applications, like treating diseases, require long-lasting changes in neural activity that persist well after the stimulation is turned off. Many studies applying tACS to humans do indeed report long-lasting EEG aftereffects, which persist for minutes to hours after stimulation [50], though similar effects have not been found in the single-unit literature [4,11–13].

While it is possible that tES reverberates through neural networks, a more likely mechanism for enduring effects is plasticity. Neural plasticity depends on the timing of neural activity, the general principle being “neurons that fire together wire together.” Changes in connectivity could therefore be produced by applying stimulation that promotes rhythmic firing, such as tACS sometimes does (Fig 1B). This appears sufficient to produce lasting changes in connectivity in vitro, but the situation is far more complicated in vivo [51], where neurons receive inputs from thousands of different sources. Intriguingly, some theories of plasticity argue that weak, sub-threshold inputs (like tES) are essential for remodeling synaptic weights [52], while synchronized oscillations also facilitate learning on very short time scales [53]. Thus, instead of attempting to precisely tune tES for individual behaviors, it may be fruitful to search for stimulation techniques that increase plasticity generally. These could then be paired with behavioral interventions for specific deficits, in the hopes of amplifying their effects. Early data is promising [48], but our ability to extend these effects is limited by our understanding of how tES affects neural activity during stimulation, when synaptic rewiring presumably occurs.

Long-lasting effects could also be produced by changing the brain’s chemistry. For example, Heimrath and colleagues found that tDCS increases inhibitory tone for 30+ minutes after stimulation [54]. Interestingly, this change in inhibition is hard to reconcile with data from isolated neurons, but it is exactly consistent with some theories of network effects (see “What about other forms of tES?”, above). These neurochemical changes could indicate altered patterns of spiking activity, but may reflect effects on non-neuronal cells; astrocytes [43] and the blood–brain barrier [55] are both affected by electric fields and the former play a critical role in synaptic plasticity.

## Why bother with tES?

It may seem as though tES is a lost cause: Its effects are neither strong, nor focal, nor easy to predict. However, many of these challenges outlined here are not specific to tES. Every form of brain stimulation, including non-invasive methods like transcranial magnetic stimulation (TMS) and focused ultrasound (fUS), as well as invasive approaches like DBS and optogenetics, must contend with ongoing neural activity. Those techniques are often so strong that they can overwhelm that activity, rather than having to compete with it. However, the brain’s ongoing dynamics also subserve important functions like the routing of information [30], which would be disrupted by stronger stimulation. The consequences of such a disruption remain unclear, but they may be as problematic as the condition the stimulation is intended to resolve. From this point of view, the relatively gentle nature of tES stimulation might be considered a positive quality. Alternately, stronger forms of stimulation may need to be carefully applied to preserve important aspects of ongoing activity, as has been proposed for TMS [56].

Another advantage of tES is that it is ready for immediate translation. Other forms of non-invasive brain stimulation require large and expensive base infrastructure: a TMS apparatus costs hundreds of thousands of dollars and requires bulky, non-portable hardware, as does fUS. Invasive approaches are also expensive, laborious, and require the patient to be in relatively good health, but for the one condition being treated. In contrast, tES devices are relatively cheap (as little as US$15) and have already been made into form factors like a baseball cap or headband that can be used outside the home during activities of daily living. Several studies have also found that patients can use tES devices independently or with support from a telehealth professional. Moreover, tES has an excellent safety record, with most non-behavioral side effects limited to mild skin irritation. Thus, it represents a flexible and easy deploy alternative to non-invasive—and certainly to invasive—approaches.

The diffuse nature of tES effects may also sometimes be beneficial. In many situations, undesirable brain states are not limited to a single region, but instead reflect a more widespread problem. This is especially true in pathological conditions: epilepsy involves excess synchrony throughout the brain; aberrant global oscillations may be implicated in the symptoms of Parkinson’s disease and depression as well. More focal techniques, like DBS, must identify specific “chokepoints” to have widespread effects throughout the brain.

In situations where the desired effects are produced by small, spatially localized groups of neurons, extremely local invasive stimulation can reliably drive behavioral effects by overpowering the brain’s ongoing activity. Stimulating a face patch, for example, can encourage an animal to see faces, even where none exist [57]. We cannot expect tES to yield the same level of power or precision, but there are relatively few situations where brain organization, on a scale of millimeters, corresponds so obviously to a behavioral goal.

## Conclusion: Torpedoes away!

When and how should we use tES is obviously the central mystery. We believe that there is now overwhelming evidence that weak electric fields, of the sort produced during tES, can have meaningful effects on the activity of neurons. There is no longer any need to evoke the magical properties of torpedo fish—or the placebo effect—as the mechanism behind tES’s apparent effects. However, the nature of those effects remains mysterious because they are so strongly shaped by interactions with the brain’s ongoing activity that the same stimulation, applied in different situations, can produce categorically different effects. The mysteries of tES are therefore intimately tied up with the mysteries of the brain itself.

We have scarcely touched on how tES affects specific behaviors. The literature is full of attempts to use tES to alter physical, cognitive, and emotional processes, but there are few direct replications and even fewer successful ones. The approaches we describe here may offer a way to develop tES interventions that produce reliable and desirable effects, but we are also limited by our understanding of the relationship between neural activity and behavior, which remains one of the largest open questions in neuroscience.

This assessment highlights our current state of ignorance about the mechanistic effects of tES, but things are not nearly as bad as they may sound. A lack of mechanistic understanding has not prevented many interventions, including aspirin, from becoming effective—or even essential—treatments [58]. Empirical studies of tES can still yield valuable data about treatments for specific conditions, and tES is easy and safe enough to apply that it may often be worth trying. However, generalizing from these data will require practitioners to reckon with the organized complexity of the brain. In the case of neural oscillations, the mathematical framework of coupled oscillators provides a principled way to do so. Future tES interventions should combine these tools with our still-growing knowledge of neural oscillations to develop methods that can robustly alter behavior across a wide range of individuals and situations.

## Supporting information

S1 Glossary A table of terms used in this article. (DOCX)

## Abbreviations

DBS: deep brain stimulation
EEG: electroencephalography
fUS: focused ultrasound stimulation
tACS: transcranial alternating current stimulation
tDCS: transcranial direct current stimulation
tES: Transcranial electrical stimulation
TMS: transcranial magnetic stimulation
tRNS: transcranial random noise stimulation

## References

[1] Kellaway P. The part played by electric fish in the early history of bioelectricity and electrotherapy. Bull Hist Med. 1946;20:112–137. 20277440

[2] Lefaucheur J-P, Antal A, Ayache SS, Benninger DH, Brunelin J, Cogiamanian F, et al. Evidence-based guidelines on the therapeutic use of transcranial direct current stimulation (tDCS). Clin Neurophysiol. 2017;128:56–92. doi: 10.1016/j.clinph.2016.10.087 27866120

[3] Schutter DJLG, Wischnewski M. A meta-analytic study of exogenous oscillatory electric potentials in neuroenhancement. Neuropsychologia. 2016;86:110–118. doi: 10.1016/j.neuropsychologia.2016.04.011 27085766

[4] Krause MR, Vieira PG, Csorba BA, Pilly PK, Pack CC. Transcranial alternating current stimulation entrains single-neuron activity in the primate brain. Proc Natl Acad Sci U S A. 2019;116:5747–5755. doi: 10.1073/pnas.1815958116 30833389 PMC6431188

[5] Saturnino GB, Siebner HR, Thielscher A, Madsen KH. Accessibility of cortical regions to focal TES: Dependence on spatial position, safety, and practical constraints. Neuroimage. 2019;203:116183. doi: 10.1016/j.neuroimage.2019.116183 31525498

[6] Vöröslakos M, Takeuchi Y, Brinyiczki K, Zombori T, Oliva A, Fernández-Ruiz A, et al. Direct effects of transcranial electric stimulation on brain circuits in rats and humans. Nat Commun. 2018;9:483. doi: 10.1038/s41467-018-02928-3 29396478 PMC5797140

[7] Lafon B, Henin S, Huang Y, Friedman D, Melloni L, Thesen T, et al. Low frequency transcranial electrical stimulation does not entrain sleep rhythms measured by human intracranial recordings. Nat Commun. 2017;8:1199. doi: 10.1038/s41467-017-01045-x 29084960 PMC5662600

[8] Asamoah B, Khatoun A, Mc LM. tACS motor system effects can be caused by transcutaneous stimulation of peripheral nerves. Nat Commun. 2019;10:266. doi: 10.1038/s41467-018-08183-w 30655523 PMC6336776

[9] Chan CY, Nicholson C. Modulation by applied electric fields of Purkinje and stellate cell activity in the isolated turtle cerebellum. J Physiol. 1986;371:89–114. doi: 10.1113/jphysiol.1986.sp015963 3701658 PMC1192712

[10] Anastassiou CA, Perin R, Markram H, Koch C. Ephaptic coupling of cortical neurons. Nat Neurosci. 2011;14:217–223. doi: 10.1038/nn.2727 21240273

[11] Ozen S, Sirota A, Belluscio MA, Anastassiou CA, Stark E, Koch C, et al. Transcranial Electric Stimulation Entrains Cortical Neuronal Populations in Rats. J Neurosci. 2010;30:11476–11485. doi: 10.1523/JNEUROSCI.5252-09.2010 20739569 PMC2937280

[12] Fröhlich F, McCormick DA. Endogenous Electric Fields May Guide Neocortical Network Activity. Neuron. 2010;67:129–143. doi: 10.1016/j.neuron.2010.06.005 20624597 PMC3139922

[13] Johnson L, Alekseichuk I, Krieg J, Doyle A, Yu Y, Vitek J, et al. Dose-dependent effects of transcranial alternating current stimulation on spike timing in awake nonhuman primates. Sci Adv. 2020;6:eaaz2747. doi: 10.1126/sciadv.aaz2747 32917605 PMC7467690

[14] Vieira PG, Krause MR, Pack CC. tACS entrains neural activity while somatosensory input is blocked. PLoS Biol. 2020;18:e3000834. doi: 10.1371/journal.pbio.3000834 33001971 PMC7553316

[15] Krause MR, Vieira PG, Thivierge J-P, Pack CC. Brain stimulation competes with ongoing oscillations for control of spike timing in the primate brain. Luo H, editor. PLoS Biol. 2022;20: e3001650. doi: 10.1371/journal.pbio.3001650 35613140 PMC9132296

[16] Huang Y, Liu AA, Lafon B, Friedman D, Dayan M, Wang X, et al. Measurements and models of electric fields in the in vivo human brain during transcranial electric stimulation. Ivry R, editor. Elife. 2017;6:e18834. doi: 10.7554/eLife.18834 28169833 PMC5370189

[17] Louviot S, Tyvaert L, Maillard LG, Colnat-Coulbois S, Dmochowski J, Koessler L. Transcranial Electrical Stimulation generates electric fields in deep human brain structures. Brain Stimul. 2022;15:1–12. doi: 10.1016/j.brs.2021.11.001 34742994

[18] Marchesotti S, Nicolle J, Merlet I, Arnal LH, Donoghue JP, Giraud A-L. Selective enhancement of low-gamma activity by tACS improves phonemic processing and reading accuracy in dyslexia. PLoS Biol. 2020;18:e3000833. doi: 10.1371/journal.pbio.3000833 32898188 PMC7478834

[19] Razza LB, Palumbo P, Moffa AH, Carvalho AF, Solmi M, Loo CK, et al. A systematic review and meta-analysis on the effects of transcranial direct current stimulation in depressive episodes. Depress Anxiety. 2020;37:594–608. doi: 10.1002/da.23004 32101631

[20] Takeuchi Y, Berényi A. Oscillotherapeutics–Time-targeted interventions in epilepsy and beyond. Neurosci Res. 2020;152:87–107. doi: 10.1016/j.neures.2020.01.002 31954733

[21] Ahn S, Mellin JM, Alagapan S, Alexander ML, Gilmore JH, Jarskog LF, et al. Targeting reduced neural oscillations in patients with schizophrenia by transcranial alternating current stimulation. Neuroimage. 2019;186:126–136. doi: 10.1016/j.neuroimage.2018.10.056 30367952 PMC6338501

[22] Alexander ML, Alagapan S, Lugo CE, Mellin JM, Lustenberger C, Rubinow DR, et al. Double-blind, randomized pilot clinical trial targeting alpha oscillations with transcranial alternating current stimulation (tACS) for the treatment of major depressive disorder (MDD). Transl Psychiatry. 2019;9:1–12. doi: 10.1038/s41398-019-0439-0 30837453 PMC6401041

[23] Pikovsky A, Kurths J, Rosenblum M, Kurths J. Synchronization: A Universal Concept in Nonlinear Sciences. Cambridge University Press; 2001.

[24] Strogatz SH. Nonlinear Dynamics and Chaos: With Applications to Physics, Biology, Chemistry, and Engineering. CRC Press; 2018.

[25] Bradley C, Nydam AS, Dux PE, Mattingley JB. State-dependent effects of neural stimulation on brain function and cognition. Nat Rev Neurosci. 2022;23:459–475. doi: 10.1038/s41583-022-00598-1 35577959

[26] Weaver W. Science and complexity. Am Sci. 1948;36:536–544. 18882675

[27] Hayek FA. The Use of Knowledge in Society. Am Econ Rev. 1945;35:519–530.

[28] Dörner D, Kimber R. The logic of failure: why things go wrong and what we can do to make them right. 1st American ed. New York: Metropolitan Books; 1996.

[29] Bergmann TO. Brain State-Dependent Brain Stimulation. Front Psychol. 2018;9. Available from: https://www.frontiersin.org/articles/10.3389/fpsyg.2018.02108. doi: 10.3389/fpsyg.2018.02108 30443236 PMC6221926

[30] Engel AK, Fries P, Singer W. Dynamic predictions: Oscillations and synchrony in top–down processing. Nat Rev Neurosci. 2001;2:704–716. doi: 10.1038/35094565 11584308

[31] Alekseichuk I, Turi Z, Amador de Lara G, Antal A, Paulus W. Spatial Working Memory in Humans Depends on Theta and High Gamma Synchronization in the Prefrontal Cortex. Curr Biol. 2016;26:1513–1521. doi: 10.1016/j.cub.2016.04.035 27238283

[32] Reinhart RMG, Nguyen JA. Working memory revived in older adults by synchronizing rhythmic brain circuits. Nat Neurosci. 2019;22:820–827. doi: 10.1038/s41593-019-0371-x 30962628 PMC6486414

[33] Haslacher D, Nasr K, Robinson SE, Braun C, Soekadar SR. Stimulation artifact source separation (SASS) for assessing electric brain oscillations during transcranial alternating current stimulation (tACS). Neuroimage. 2021;228:117571. doi: 10.1016/j.neuroimage.2020.117571 33412281 PMC7903161

[34] Noury N, Siegel M. Analyzing EEG and MEG signals recorded during tES, a reply. Neuroimage. 2018;167:53–61. doi: 10.1016/j.neuroimage.2017.11.023 29155079

[35] Doelling KB, Assaneo MF. Neural oscillations are a start toward understanding brain activity rather than the end. PLoS Biol. 2021;19:e3001234. doi: 10.1371/journal.pbio.3001234 33945528 PMC8121326

[36] Kopell N, Ermentrout GB, Whittington MA, Traub RD. Gamma rhythms and beta rhythms have different synchronization properties. Proc Natl Acad Sci U S A. 2000;97:1867–1872. doi: 10.1073/pnas.97.4.1867 10677548 PMC26528

[37] Jensen O, Goel P, Kopell N, Pohja M, Hari R, Ermentrout B. On the human sensorimotor-cortex beta rhythm: Sources and modeling. Neuroimage. 2005;26:347–355. doi: 10.1016/j.neuroimage.2005.02.008 15907295

[38] Ali MM, Sellers KK, Fröhlich F. Transcranial Alternating Current Stimulation Modulates Large-Scale Cortical Network Activity by Network Resonance. J Neurosci. 2013;33:11262–11275. doi: 10.1523/JNEUROSCI.5867-12.2013 23825429 PMC6618612

[39] Weerasinghe G, Duchet B, Cagnan H, Brown P, Bick C, Bogacz R. Predicting the effects of deep brain stimulation using a reduced coupled oscillator model. Rubin J, editor. PLoS Comput Biol. 2019;15:e1006575. doi: 10.1371/journal.pcbi.1006575 31393880 PMC6701819

[40] Datta A, Bansal V, Diaz J, Patel J, Reato D, Bikson M. Gyri–precise head model of transcranial DC stimulation: Improved spatial focality using a ring electrode versus conventional rectangular pad. Brain Stimul. 2009;2:201–207. doi: 10.1016/j.brs.2009.03.005 20648973 PMC2790295

[41] Murthy VN, Fetz EE. Effects of Input Synchrony on the Firing Rate of a Three-Conductance Cortical Neuron Model. Neural Comput. 1994;6:1111–1126. doi: 10.1162/neco.1994.6.6.1111

[42] Krause MR, Zanos TP, Csorba BA, Pilly PK, Choe J, Phillips ME, et al. Transcranial Direct Current Stimulation Facilitates Associative Learning and Alters Functional Connectivity in the Primate Brain. Curr Biol. 2017;27:3086–3096.e3. doi: 10.1016/j.cub.2017.09.020 29033331

[43] Monai H, Ohkura M, Tanaka M, Oe Y, Konno A, Hirai H, et al. Calcium imaging reveals glial involvement in transcranial direct current stimulation-induced plasticity in mouse brain. Nat Commun. 2016;7:11100. doi: 10.1038/ncomms11100 27000523 PMC4804173

[44] Márquez-Ruiz J, Leal-Campanario R, Sánchez-Campusano R, Molaee-Ardekani B, Wendling F, Miranda PC, et al. Transcranial direct-current stimulation modulates synaptic mechanisms involved in associative learning in behaving rabbits. Proc Natl Acad Sci U S A. 2012;109:6710–6715. doi: 10.1073/pnas.1121147109 22493252 PMC3340065

[45] Ozeki H, Finn IM, Schaffer ES, Miller KD, Ferster D. Inhibitory stabilization of the cortical network underlies visual surround suppression. Neuron. 2009;62:578–592. doi: 10.1016/j.neuron.2009.03.028 19477158 PMC2691725

[46] Sanzeni A, Akitake B, Goldbach HC, Leedy CE, Brunel N, Histed MH. Inhibition stabilization is a widespread property of cortical networks. O’Leary T, Huguenard J, Adesnik H, editors. Elife. 2020;9: e54875. doi: 10.7554/eLife.54875 32598278 PMC7324160

[47] Liu LD, Miller KD, Pack CC. A Unifying Motif for Spatial and Directional Surround Suppression. J Neurosci. 2018;38:989–999. doi: 10.1523/JNEUROSCI.2386-17.2017 29229704 PMC5783971

[48] Herpich F, Melnick MD, Agosta S, Huxlin KR, Tadin D, Battelli L. Boosting Learning Efficacy with Noninvasive Brain Stimulation in Intact and Brain-Damaged Humans. J Neurosci. 2019;39:5551–5561. doi: 10.1523/JNEUROSCI.3248-18.2019 31133558 PMC6616291

[49] Pavan A, Ghin F, Contillo A, Milesi C, Campana G, Mather G. Modulatory mechanisms underlying high-frequency transcranial random noise stimulation (hf-tRNS): A combined stochastic resonance and equivalent noise approach. Brain Stimul. 2019;12:967–977. doi: 10.1016/j.brs.2019.02.018 30833217

[50] Veniero D, Vossen A, Gross J, Thut G. Lasting EEG/MEG Aftereffects of Rhythmic Transcranial Brain Stimulation: Level of Control Over Oscillatory Network Activity. Front Cell Neurosci. 2015;9:477. doi: 10.3389/fncel.2015.00477 26696834 PMC4678227

[51] Yao H, Dan Y. Stimulus Timing-Dependent Plasticity in Cortical Processing of Orientation. Neuron. 2001;32:315–323. doi: 10.1016/s0896-6273(01)00460-3 11684000

[52] Magee JC, Grienberger C. Synaptic Plasticity Forms and Functions. Annu Rev Neurosci. 2020;43:95–117. doi: 10.1146/annurev-neuro-090919-022842 32075520

[53] Csorba BA, Krause MR, Zanos TP, Pack CC. Long-range cortical synchronization supports abrupt visual learning. Curr Biol. 2022;32: 2467–2479.e4. doi: 10.1016/j.cub.2022.04.029 35523181

[54] Heimrath K, Brechmann A, Blobel-Lüer R, Stadler J, Budinger E, Zaehle T. Transcranial direct current stimulation (tDCS) over the auditory cortex modulates GABA and glutamate: a 7 T MR-spectroscopy study. Sci Rep. 2020;10:20111. doi: 10.1038/s41598-020-77111-0 33208867 PMC7674467

[55] Shin DW, Fan J, Luu E, Khalid W, Xia Y, Khadka N, et al. In Vivo Modulation of the Blood–Brain Barrier Permeability by Transcranial Direct Current Stimulation (tDCS). Ann Biomed Eng. 2020;48:1256–1270. doi: 10.1007/s10439-020-02447-7 31916126 PMC7096245

[56] Romei V, Thut G, Silvanto J. Information-Based Approaches of Noninvasive Transcranial Brain Stimulation. Trends Neurosci. 2016;39:782–795. doi: 10.1016/j.tins.2016.09.001 27697295

[57] Afraz S-R, Kiani R, Esteky H. Microstimulation of inferotemporal cortex influences face categorization. Nature. 2006;442:692–695. doi: 10.1038/nature04982 16878143

[58] Filmer HL, Mattingley JB, Dux PE. Modulating brain activity and behaviour with tDCS: Rumours of its death have been greatly exaggerated. Cortex. 2020;123:141–151. doi: 10.1016/j.cortex.2019.10.006 31783223

