# Supplementary material for: Transcranial electrical stimulation: How can a simple conductor orchestrate complex brain activity?
Source: PLoS Biol. 2023 Jan 30;21(1):e3001973. doi: 10.1371/journal.pbio.3001973 (PMC9886255; doi:10.1371/journal.pbio.3001973)
Supplement: S1 Glossary — (DOCX) [file pbio.3001973.s001.docx]

| **Anode** | The point where current enters a system |
| --- | --- |
| **Cathode** | The point where current leaves a system |
| **Closed-loop system** | A system that uses feedback from the controlled object (e.g., the brain) to alter its control strategy. See “open-loop”. |
| **Deep brain stimulation (DBS)** | Invasive brain stimulation performed with electrodes implanted in deep (subcortical) structures. Often used clinically in the treatment of Parkinson’s Disease |
| **Electroencephalogram, electroencephalography (EEG)** | Non-invasive recordings of bulk brain activity made using electrodes placed on the intact scalp. |
| **Focused ultrasound stimulation (fUS)** | Non-invasive brain stimulation using high-intensity sound waves to excite neurons. |
| **Open-loop system** | A system that uses a pre-defined control strategy that is executed without reference to the controlled object’s state. |
| **Optogenetics** | The introduction of light-sensitive proteins into cells, allowing precise control of their activity. |
| **Transcranial magnetic stimulation (TMS)** | Non-invasive brain stimulation using very strong pulsed magnetic fields, which induce electrical currents in neurons. |
